# ASM is a therapeutic target in dermatomyositis by regulating the differentiation of naive CD4 + T cells into Th17 and Treg subsets

**DOI:** 10.1186/s13395-024-00347-1

**Published:** 2024-07-18

**Authors:** Yuehong Chen, Huan Liu, Zhongling Luo, Jiaqian Zhang, Min Dong, Geng Yin, Qibing Xie

**Affiliations:** 1 Department of Rheumatology and Immunology, West China Hospital, Sichuan University, 37 Guoxue lane, Chengdu, 610041 China; 2 Department of General Practice, West China Hospital, General Practice Medical Center, Sichuan University, 37 Guoxue lane, Chengdu, 610041 China

**Keywords:** Dermatomyositis, Acid sphingomyelinase, CD4 T cell, Th17, Treg cells

## Abstract

**Background:**

This study aims to investigate the involvement of acid sphingomyelinase (ASM) in the pathology of dermatomyositis (DM), making it a potential therapeutic target for DM.

**Methods:**

Patients with DM and healthy controls (HCs) were included to assess the serum level and activity of ASM, and to explore the associations between ASM and clinical indicators. Subsequently, a myositis mouse model was established using ASM gene knockout and wild-type mice to study the significant role of ASM in the pathology and to assess the treatment effect of amitriptyline, an ASM inhibitor. Additionally, we investigated the potential treatment mechanism by targeting ASM both in vivo and in vitro.

**Results:**

A total of 58 DM patients along with 30 HCs were included. The ASM levels were found to be significantly higher in DM patients compared to HCs, with median (quartile) values of 2.63 (1.80–4.94) ng/mL and 1.64 (1.47–1.96) ng/mL respectively. The activity of ASM in the serum of DM patients was significantly higher than that in HCs. Furthermore, the serum levels of ASM showed correlations with disease activity and muscle enzyme levels. Knockout of ASM or treatment with amitriptyline improved the severity of the disease, rebalanced the CD4 T cell subsets Th17 and Treg, and reduced the production of their secreted cytokines. Subsequent investigations revealed that targeting ASM could regulate the expression of relevant transcription factors and key regulatory proteins.

**Conclusion:**

ASM is involved in the pathology of DM by regulating the differentiation of naive CD4 + T cells and can be a potential treatment target.

**Supplementary Information:**

The online version contains supplementary material available at 10.1186/s13395-024-00347-1.

## Introduction

Dermatomyositis (DM) is a subtype of idiopathic inflammatory myopathy characterized by skin rashes like heliotrope rash or Gottron sign, symmetric proximal limb muscle weakness, and the presence of myositis-specific antibodies including anti-MDA5, Anti-Mi2, antiNXP2, anti-TIF1, among others. These antibodies aid in classifying clinical features and guiding management [1, 2]. The reported prevalence of DM ranges from 1 to 6 per 100,000 in the United States and 3.8 per 100,000 in Sweden, with a higher prevalence among individuals aged 50 to 79 years and a two-fold higher occurrence in females compared to males [2, 3]. Apart from affecting muscles and skin, DM can also involve in the immune system, lungs, heart, gastrointestinal tract, endocrine system and other tissues, which greatly affect the diagnosis and treatment as well as intensify the financial burden [2]. Following a DM diagnosis, screening for malignancies and interstitial lung disease is recommend due to the high risk of developing malignancies with a prevalence of 20% and interstitial lung disease with a prevalence of 42% [1, 4]. Pathologically, DM is characterized by perifascicular atrophy and the infiltration of various immune cells like macrophages, B cells, T cells, plasma cells, and plasmacytoid dendritic cells [2].Currently, the primary treatment options for DM involve a combination of glucocorticoids and immunosuppressive drugs like methotrexate, azathioprine, calcineurin inhibitors, and mycophenolate mofetil, among others. However, these treatments are associated with adverse effects such as an increased risk of infection, bone marrow suppression, liver toxicity, osteoporosis, and a high recurrence rate, which limits their use [5–7]. Recent advancements in understanding the pathological mechanism of DM have identified potential targets like T cells, B cells, cytokines such as tumor necrosis factor-α (TNF-α), and Janus kinase (JAK) for the treatment. Although targeted treatments have shown promise, the evidence supporting their effectiveness primarily comes from case reports or case series, highlighting the need for more high-quality studies [8–13]. The current immunosuppressive drugs have limited efficacy, and addressing the key pathological molecules involved in DM still falls short of meeting therapeutic demands. Therefore, the identification of new therapeutic targets has become a pressing matter. While the exact mechanism behind DM remains unclear, the search for novel key regulatory molecules offers hope for future treatments.

Acid sphingomyelinase (ASM) is an enzyme that breaks down lipids present in cell membrane and lysosomes. It also interacts with various proteins distributed throughout the cell membrane [14]. ASM can hydrolyze the phosphodiester bonds of sphingomyelin on the plasma membrane, leading to the generation of ceramides. This enzymatic function of ASM is crucial for controlling cellular immune responses, as well as cellular processes like differentiation, proliferation, and apoptosis [15, 16]. It is worth noting that the sphingomyelin/ceramide signaling pathway, regulated by ASM, has been implicated in various diseases such as cancer [17], cardiovascular diseases like atherosclerosis [18], neurological disorders including Alzheimer’s disease [19], and respiratory conditions like chronic obstructive pulmonary disease [20]. Consequently, this pathway represents a promising target for therapeutic intervention in these conditions. However, the specific role of ASM in the pathology of DM remains incompletely understood. This study presents evidence linking ASM to the pathology of DM using a well-established mouse model, experimental autoimmune myositis (EAM) mouse model to study myositis. Additionally, it demonstrates that ASM plays a crucial role in regulating the differentiation of naive CD4 + T cells into distinct T cell subsets, suggesting ASM as a potential therapeutic target for myositis treatment.

## Methods

### Reagents

RPMI-1640 media (abs9468) was purchased from univ bio-technology CO., Ltd (Shanghai, China) and fetal bovine serum (FBS, 10099-141) was purchased from Gibco (Grand Island, NY, USA). Complete Freund’s adjuvant (7027) and incomplete Freund’s Adjuvant (7002) was purchased from Chondrex (Chondrex Inc, WA, USA). Amitriptyline hydrochloride (AMI, HY-B0527A) was purchased from MedChemExpress (Monmouth Junction, NJ, USA). Plastic feeding tubes (TFEP-001, 2.25 × 50 mm) were purchased from Shanghai Yuyan Instruments Company (Shanghai, China). The RNeasy^®^ Mini kit (74,104) was purchased from Qiagen (Hilden, Germany). The anti-GAPDH antibody (EPR16891, ab181602), anti-Acid sphingomyelinase antibody (ab83354), acidic Sphingomyelinase Assay Kit (Fluorometric) (ab190554), and human Acid sphingomyelinase ELISA Kit (SMPD1) (ab277075) were purchased from Abcam (Cambridge, UK). ChamQ SYBR qPCR Master Mix (Q311-02) and HiScript^®^ III RT SuperMix for qPCR (+ gDNA wiper, R323-01) were purchased from Vazyme (Nanjing, China). The anti-STAT3 antibody (AF6294), anti-STAT3 (phospho Y705) antibody (AF3293), and enhanced chemiluminescence kit (KF005) were purchased from Affinity Biosciences (Cincinnati, OH, USA). The Immobilon^®^-P transfer membrane, 0.45 μm (IPVH00010), was purchased from Merck Millipore (Billerica, MA, USA). The anti-STAT5 Antibody (381,427) and phospho-STAT5 (Tyr694, 381,125 ) were bought from ZEN BIO (Shanghai, China). Mouse IL-6 ELISA kit (MM-1011M1), mouse IL-10 ELISA kit (MM-0176M1), mouse IL-17 A ELISA kit (MM-0759M1), mouse ASM ELISA kit (MM-46150M1) were bought from Meimian (Jiangsu, China). Cell lysis buffer (P0013) and the BCA protein quantitation assay (P0010) were purchased from Beyotime (Shanghai, China). The protease inhibitor cocktail (GK10014), phosphatase inhibitor cocktail I (GK10011), phosphatase inhibitor cocktail II (GK10012), and bordetella pertussis toxin (GC17532) were purchased from Glpbio (Montclair, CA, USA). PBS (1×, G4202) and environmentally friendly GD fixing solution (G1111-100mL) were purchased from Servicebio (Wuhan, China). Foxp3 / Transcription Factor Staining Buffer Set (00-5523-00), ic fixation buffer (00-822-49), fixable Viability Dye 780 APC-cy7 (50-169-66), and permeabilization Buffer (00-8333-56) were bought from ThermoFisher Scientific (Waltham, MA, USA). EasySep™ Mouse Naïve CD4 + T Cell Isolation Kit (19,765) were bought from Stemcell Technologies (Canada). Mouse Th17 Cell Differentiation Kit (CDK017) was bought from R&D Systems (minneapolis, minnesota, USA). Anti-Mouse CD3 BV510 (740,147), anti-Mouse CD25 BB515 (564,424), anti-Mouse FOXP3 APC (560,401), and GolgiStop™ Protein Transport Inhibitor (554,724) were bought from BD Biosciences (New Jersey, USA). Anti-Mouse CD4 Percp-cy5.5 (E-AB-F1097J) and anti-Mouse IL-17 A PE (E-AB-F1199D) were bought from Elabscience (Wuhan, China). Plant hemagglutinin (48–68) (115721-95-4) was bought from AbMole (Chicago, USA). Grip Strength Meter (yls-13 A) was bought from Jinan Yiyan Technology Development Co., LTD (Jinan, China).

### Study population

Patients who met the EULAR/ACR criteria for the diagnosis of DM [21] Were recruited tetween December 2019 and November 2020 at the Department of Rheumatology, West China Hospital of Sichuan University. The exclusion criteria were as follows: other lung diseases such as idiopathic pulmonary fibrosis, pulmonary sarcoidosis, pulmonary infection, and chronic obstructive pulmonary disease; autoimmune diseases other than DM; malignant diseases; pregnancy; and overall poor health. A total of 58 DM patients and 30 healthy controls (HCs) matched for age and gender were included. The detailed information regarding the enrolled DM patients and HCs is provided in Table 1. This study was conducted in compliance with the Declaration of Helsinki and approved by the ethics committee of West China Hospital (No. 246 in 2019). Written informed consent was obtained from all participants, and all methods followed relevant guidelines and regulations.

**Table 1:** Characteristics of included patients

	HC	DM
Patients (n)	30	58
Age (mean ± SD, years)	47.73 ± 7.64	48.16 ± 9.52
Gender# (Female NO., %)	26, 83.33	42, 72.41
Type of antibodies (NO., %)		
anti MDA-5 anityboby		26, 44.83
anti Mi-2 antibody		3, 5.17
anti NXP2 antibody		3, 5.17
anti SRP antibody		3, 5.17
anti SAE antibody		3, 5.17
anti HMGCR antibody		1, 1.72
anti TIF1 antibody		1, 1.72
Treatments (NO, %)		
Glucocorticoids		58, 100
Hydroxychloroquine		38, 65.52
Cyclophosphamide		23, 39.66
Methotrexate		17, 29.31
Tacrolimus		12, 20.69
Mycophenolate mofetil		4, 6.90
Cyclosporin		4, 6.90

### Myositis disease activity assessment

During the clinical evaluation, two experienced rheumatologists collected data on the patients’ medical history and conducted physical examinations. The myositis disease activity assessment tool [22] was used to measure disease activity, specifically utilizing the myositis intention to treat activity index (MITAX). The global disease activity score derived from MITAX is computed by summing as the total of the worst category scores for each of the seven individual organ systems (constitutional, cutaneous, skeletal, gastrointestinal, pulmonary, cardiac, and muscle). This score is divided by the maximum possible score, which ranges from 1 to 63. Each organ system is categorized into five categories: A (active), B (beware), C (contentment), D (discount), and E (no evidence). These categories correspond to values of 9, 3, 1, 0 (indicating no current activity but previously active), and 0 (indicating no current or previous activity), respectively. Therefore, a higher score indicates increased disease activity. The physician assessed disease activity at the time of enrollment.

### Cutaneous damage assessment

The cutaneous assessment tool-binary method (CAT-BM) [23] was utilized to assess cutaneous damage with disease activity evaluate based on 7 indicators: Gottron papule, Heliotrope rash, erythema on the zygomatic or facial regions, linear erythema on the limbs’ extension side, V-zone erythema on the front of the neck, erythema on the back of the neck and shoulder (known as the Shawl sign), and erythema on non-exposed areas. Disease activity scores ranged from 0 to 17 while lesion severity was scored on a 0–11 scale, accounting for atrophy or pigmentation at the lesion site. The total score, ranging from 0 to 28, was calculated by combining the disease activity and lesion severity scores.

### Detection of serum ASM levels

Each patient and HC provided a 4mL venous blood sample, which was collected into a coagulating agent-containing blood collection tube. The samples were then allowed to clot at room temperature for about 2 h. After clot formation, the samples were centrifuged at 2000 g for 10 min. Subsequently, the sera were collected and stored at a temperature of − 80° C. Human ASM ELISA kit was utilized to assess serum ASM levels following the kit’s provided specifications.

### Mice

Female and male C57BL/6J-smpd1 heterozygous mice, aged four to six weeks, were obtained from Cyagen Bioscience (Guangzhou, China) and were then bred to generate SMPD1^−/−^ mice. Wild-type C57BL/6 female mice, aged seven weeks, were obtained from the Beijing Huafukang Biotechnology Company (Beijing, China). The mice were randomly housed in cages with five mice per cage at the Animal Facility of Chengdu Frontier Medical Center, West China Hospital, Sichuan University, under pathogen-free conditions. They were provided with adequate food and water and allowed to acclimate for one week before any experiments were conducted. The mice were maintained on a 12-hour light and 12-hour dark cycle at a consistent temperature ranging from 22 to 24 ◦C. Seven-week-old female wild-type guinea-pigs were purchased from Byrness Weil Biotech Ltd. (Chongqing, China). All animal experiments performed in this study were approved by the Animal Ethics Committee of West China Hospital, Sichuan University (approval number: 2,020,243 A).

### Genotyping strategy

Genotyping of SMPD1^−/−^ mice was conducted following protocols provided by Cyagen Bioscience. In brief, tails from 3-week-old mice were digested in a buffer composed of 50 mM KCl, 10 mM Tris-HCl (pH 9.0), 0.1% Triton X-100, and 0.4 mg/mL Proteinase K. Then, the genomic DNA were amplified with the following components: 7.9 µL of double-distilled water, 0.8 µL of forward primer, 0.8 µL of reverse primer, 10 µL of 2 ×Mouse Direct PCR Mix and 0.5 µL of DNA. This amplification process involved 35 cycles. The PCR conditions consisted of denaturation at 94 ◦C for 5 min, annealing at 58 ◦C for 30 s, extension at 72 ◦C for 30 s per kilobase pair, and an additional extension at 72 ◦C for 7 min. Then, PCR products were visualized by agarose gel electrophoresis. The primer sequences employed are listed below: PCR primer set 1, F1: 5′ -GCA AAG TCT TAT TCA CTG CTC T-3′, R1:5′ -AGA GAT GTT CCA AGT CGA AAA GAT-3′, product size: 641 bp; PCR primer set 2, F1: 5′ -TAA AGT TAG GGA GAG TAA AGT CAG C-3′, R2: 5′ -CCA TCT ATT TGG TAA ACT CGG TAG-3′, product size: 550 bp.

### EAM mouse model

After a week of housing and acclimatization at the animal facility, mice were randomly assigned to each group using a random number table, with six mice in each group, to establish the EAM model. The model was induced by 1.5 mg of myosin extracted from wild-type guinea pigs, along with complete Freund’s adjuvant containing 10 mg/ml of Mycobacterium tuberculosis. This method was based on previously published protocols [24]. Drugs were administered daily through oral gavage two days prior to model establishment and continued until the end of the experiment. The model group received a drug dilution buffer composed of water, ethanol, and 2% acetic acid in a ratio of 8:3:1 by volume, with less than 5% of DMSO included. The drug groups were administrated the same concentrations of DMSO and dilution buffer, along with varying concentrations of the tested drugs (AMI 1 mg/kg or 10 mg/kg).

At the conclusion of the study, mice were subjected to muscle strength testing using a mouse grip tester (YLS-13 A, Jinan Yiyan Technology Development Co., LTD, China). The test was repeated three times, and the average values were recorded. Subsequently, the mice were sacrificed and blood samples were collected to obtain sera. The spleens were weighed and used for flow cytometry analysis to detect the CD4 T cell subsets Th17 and Treg. Moreover, samples of the gastrocnemius muscle were collected and either preserved as fresh specimens in liquid nitrogen or fixed in the GD fixing solution for pathological analysis.

### Detection of serum CK

Mouse serum samples were sent to Wuhan Servicebio Technology Co., Ltd (Wuhan, China) for the detection of serum CK levels. The analysis was performed using an automatic biochemistry analyzer (Chemray 800) manufactured by Rayto Life and Analytical Sciences Co., Ltd ( Shenzhen, China ).

### Flow cytometry

To detect the percentages of CD4 T subsets in spleens, we utilized the following fluorescent-labeled antibodies: APC-Cy7-FVS780 (1:2000) for cell death, Anti-Mouse CD3 BV510 (1:50), Anti-Mouse CD4 Percp-cy5.5 (1:50), Anti-Mouse CD25 BB515 (1:50), Anti-Mouse IL-17 A PE (1:50), and Anti-Mouse FOXP3 APC (1:50) for Th17 or Tregs. The stained samples were analyzed using a flow cytometer (Cytoflex, Beckmam, USA).

### Muscle homogenate

To prepare the muscle homogenates, the muscle was removed from liquid nitrogen and placed on ice for thawing. The muscle tissues were then fragmented into small sections and transferred to tissue grinding tubes, each containing 1 large steel ball (4 mm in diameter) and 2 small steel balls (3 mm in diameter) for grinding. Samples were obtained by adding PBS with a protease inhibitor (at a ratio of 1:100) to the tubes With each tube having 9 µL of PBS per 1 mg of tissue. The low temperature grinder (KZ-III-FP, Servicebio) was pre-chilled before use. The muscle pieces were ground for 10 s at of 70 Hz with three cycles of grinding at -10 °C and a 20 s pause between each cycle. After grinding, the samples were stored at -20 °C overnight, then thawed twice in liquid nitrogen and centrifuged at 5000 *g* for 10 min at 4 °C. The resulting supernatants were collected and the protein concentrations were determined using a BCA protein concentration assay kit.

### H&E staining

Muscle tissues were fixed in an environmentally friendly solution, GD fixing solution, for 24 h. Subsequently, the tissues underwent paraffin embedding and sectioning to produce 5 μm sections. The hematoxylin and eosin (H&E) staining procedure was conducted following the manufacturer’s instructions. Neutral gum was applied to seal the slides which were subsequently stored at room temperature. An automated quantitative pathology imaging system was employed to scan the stained sections (Vectra Polaris, United States).

### Quantitative analysis of H&E staining

Pathological qualitative scores were assessed based on the infiltration of inflammatory cells in the H&E stained sections of mouse muscles [25]. The scoring system ranged from 0 to 4.5, with scores of 1 indicating less than 5 muscle fibers affected, scores of 2 indicating 5 to 30 muscle fibers involved, scores of 3 indicating the involvement of a muscle bundle, and scores of 4 representing diffused widespread lesions. Additionally, a score of 0.5 was added when multiple lesions were found in a muscle segment.

### IHC staining

To analyze the levels of crucial proteins, we employed immunohistochemical (IHC) staining.The process began with dewaxing the slides using xylene followed by hydration with gradient alcohol. Subsequently, antigen retrieval was performed, along with blocking of endogenous peroxidase and non-specific binding sites. The target protein was then detected using a primary antibody (diluted at 1:100) and the slides were left to incubate overnight at 4 ℃. The next day, thorough washing of the slides was carried out, followed by the addition of a secondary antibody labeled with horse radish peroxidase (diluted at 1:500) and an hour-long incubation at room temperature. A 3,3’-diaminobenzidine (DAB) kit was used to develop a brown color,, cell nuclei were counterstained, and the slides were sealed. Finally, an automatic quantitative pathology imaging system was employed to automatically scan the slides (Vectra Polaris, USA).

### ELISA

Enzyme-linked immunosorbent assay (ELISA) kits were employed according to the manufacturer’s instructions to measure the levels of ASM, IL-6, IL-10, and IL-17 A in the sera or tissue homogenates of mice. The optical density at 450 nm was measured using a microplate reader (CLARIOstar, BMG LABTECH, Germany), and protein concentrations were determined via a standard curve.

### qRT-PCR

To analyze relative target gene expressions, the method of quantitative reverse transcriptase polymerase chain reaction (qRT-PCR) was employed. The expressions of target genes were normalized with the internal reference GAPDH. The collection of RNAs from either cells or tissues was carried out following the instructions of the RNA extraction kit. The extracted RNA was then reverse transcribed into complementary DNA (cDNA), which served as the template for synthesizing the target gene using the specific primers. The quantification of gene expression levels was determined using the formula 2^−ΔΔCq^. The following set of primers was utilized for the experiment: GAPDH primers for human (hGAPDH): F 5′-3′: CAC ATG GCC TCC AAG GAG TAA, R 5′-3′: TGA GGG TCT CTC TCT TCC TCT TGT; hASM: F 5′-3′: CTG TCT GAC TCT CGG GTT CTC, R 5′-3′: CTA TGC GAT GTA ACC TGGCAG; GAPDH primers for mouse (mGAPDH): F 5′-3′: AGG TCG GTG TGA ACG GAT TTG, 5′-3′ R: GGG GTC GTT GAT GGC AAC A; mIL-6: F 5′-3′: TTC CAT CCA GTT GCC TTC TTG, R 5′-3′: AGG TCT GTT GGG AGT GGT ATC; mIL-10: F 5′-3′: CTT ACT GAC TGG CAT GAG GAT CA, R 5′-3′: GCA GCT CTA GGA GCA TGT GG; mIL-17: 5′-3′ F: TCA GCG TGT CCA AAC ACT GAG, 5′-3′ R: CGC CAA GGG AGT TAA AGA CTT; mFOXP3: 5′-3′ F: AGT GGC AGG GAA GGA GTG TCA G, 5′-3′ R: AGG CTG GAT AAC GGC AGA GGA G; mRORγT: 5′-3′ F: AAG GTG GTA CTG GGT ATG GC, 5′-3′ R: CTC TTG GGC CTT GCA GTC TT; mASM: 5′-3′ F: ACT CCA CGG TTC TTT GGG TTC, 5′-3′ R: CGG CGC TAT GGC ACT GAA T.

### Western blotting

Jurkat T cells were cultivated in RPMI-1640 medium supplemented with 10% fetal bovine serum and 1% penicillin-streptomycin, incubated at 37 ℃ with 5% CO2. Cell passages were performed if the density exceeded 1.0 × 10^6^ cells per milliliter. For key proteins analysis in the cell signaling pathway, Jurkat T cells were seeded in 6-well plates at a density of 5 × 10^4^ cells per milliliter. AMI at 1µM or 10 µM was added, followed by overnight incubation. The next day, cells were stimulated with plant hemagglutinin at 5 µg/mL for 30 min before proteins extraction.

Protein expression levels were detected using Western blotting (WB). Protein extraction was carried out from stimulated cells or protein supernatants from muscle homogenates. Following determination of the protein concentrations, denaturation was achieved by adding loading buffer and heating at 95 °C for 10 min. Subsequently, 20 µg of proteins were loaded onto a gel for separation via gel electrophoresis. Protein transfer onto a nitrocellulose membrane was employed using a wet transfer system. The membrane was then blocked at room temperature for 30 min using 5% (w/v) non-fat milk in 1×Tris Buffered Saline containing 1‰ Tween 20 (TBST). Primary antibody was added to the membrane and left overnight at 4 °C. After three washes with TBST, the membrane was exposed to the secondary antibody for 1 h at ambient temperature. Following additional washes, the bands on the membrane were visualized by gel scanner (ChemiDoc XRS, BIO-RAD, USA) with an enhanced chemiluminescent substrate.

### Spleen naïve CD4 + T cell differentiation

The differentiation of naïve CD4 + T cells from the spleen into T cell subsets was conducted using established methodologies [26, 27]. For Th17 differentiation, a 24-well plate was coated with 1 mL of PBS containing anti-CD3 (2 µg/mL) and anti-CD28 (1 µg/mL) and incubated overnight at 4 °C. The following day, spleens from 8-week-old WT C57BL/6 mice were harvested. Spleen cells were isolated by grinding the spleen on a 70-µm mesh and collected in PBS supplemented with 1% FBS. The collected cells were centrifuged at 1000 rpm for 5 min, repeated twice, and red blood cells were lysed with ACK lysing buffer for 5 min on ice. The CD4 + T cells were then isolated using a CD4 + T cell isolation kit following the manufacturer’s instructions. The coating buffer was removed, and the plate was washed with PBS before seeding the CD4 + T cells in the Th17 differentiation buffer. Additionally, AMI (1µM) or AMI (10µM) was added. After four days, the cells were haevested for flow cytometry analysis. To assess differentiation, 1 µL of Golgi stop buffer was added to each well and incubated for 4 h. Subsequently, the cells were stained. Cell death was assessed using APC-Cy7-FVS780 (1:2000), and Th17 cells were stained with Anti-Mouse CD3 BV510 (1:50), Anti-Mouse CD4 Percp-cy5.5 (1:50), and Anti-Mouse IL-17 A PE (1:50). Data acquisition was performed using a flow cytometry instrument (Cytoflex, Beckmam, USA).

### Statistical analysis

The data analysis software SPSS version 22.0 (SPSS, Inc., Chicago, IL, USA) or GraphPad Prism version 6.0 (GraphPad, Inc., La Jolla, CA, USA) was utilized for organizing all data. The normal distribution of continuous variables was assessed using the Kolmogorov-Smirnov test. Data were presented as either the mean ± standard deviation (SD), median (quartile), or number (percentage). The Mann-Whitney U-test was employed to compare differencies between the two groups of continuous variables with non-normal distribution. The t-test was utilized to compare two means, while one-way ANOVA was employed for comparisons among more than two means. Bonferroni corrections were applied for pairwise comparisons among multiple groups. Spearman’s correlation coefficient (r) was employed to analyze the relationships between serum ASM levels and myositis disease activity, activity and damage of cutaneous manifestations, muscle enzymes including CK, LDH, HBDH, AST, and other clinical indicators. Statistical significance was set at a p value of less than 0.05. Any result with a p value less than 0.05 was considered statistically significant, denoted by asterisks(* < 0.05, ** < 0.01, *** < 0.001, **** < 0.0001).

## Results

### ASM is highly expressed in DM and associates with disease activity

**Fig. 1:**
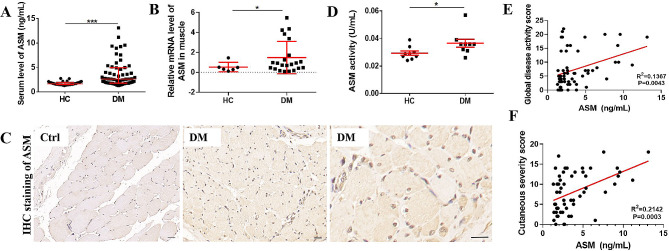
ASM exhibits high expression in DM patients and associates with disease activity. (**A**) serum ASM levels detected using ELISA (**B**) mRNA expression level of ASM in muscle tissues of patients (**C**) protein levels of ASM in patients’ muscle tissues, scale bar 20 μm (**D**) serum ASM activity (**E**) Correlation analysis between serum ASM levels and overall disease activity (**F**) Correlation analysis between serum ASM levels and cutaneous severity score. *P < 0.05, **P < 0.01, ***P < 0.001, *n* = 30 in healthy controls, *n* = 58 in patients with DM

A total of 58 DM patients were included in the study, with an average age of 48.16 ± 9.52 years, along with 30 HCs with an average age of 47.73 ± 7.64 years. Among the DM patients, 72.41% were female, whereas among the HCs, 83.33% were female. There were no statistically differences in age (*p* = 0.834) and gender (*p* = 0.130) between the DM patients and HCs (Table 1). To assess the expression levels of ASM in DM, the serum ASM levels were measures. The results indicated a significant increase of ASM levels in DM patients compared to HCs, with median values of 2.63 (1.80–4.94) ng/mL and 1.64 (1.47–1.96) ng/mL, respectively (Fig. 1A). Subgroup analysis by gender revealed that gender did not have a significant impact on serum ASM levels in both DM patients, female 2.51 (1.79–3.95) ng/mL versus male 2.73 (2.01–5.50) ng/mL, *p* = 0.3121, and HCs, female 1.71 (1.47-2.00) ng/mL versus male 1.53 (1.52–1.55) ng/mL, *p* = 0.209. Subsequently, the expression levels of ASM in muscle tissue were examined using qRT-PCR and IHC, showing increased levels in DM patients compared to HCs. Particularly, ASM was predominantly expressed in infiltrated nucleated cells at the perimysium, both in the cytoplasm and nuclei, as well as on plasma membrane (Fig. 1B-C). Additionally, the activity of ASM in the serum of DM patients was found to be significantly higher than in HCs (Fig. 1D). Thus, both the activity and levels of ASM are notably elevated in patients with DM.

To investigate the relationship between serum levels of ASM and clinical disease activity as well as clinical laboratory indicators, we conducted correlation analysis. The results revealed a correlation between ASM levels and both the global disease activity score (R^2^ = 0.1367, *p* = 0.0043) and cutaneous severity score (R^2^ = 0.2142, *p* = 0.0003) (Fig. 1E-F). Additionally, we observed associations between ASM levels and muscle enzymes, such as LDH (R^2^ = 0.1663, *p* = 0.0016), HBDH (R^2^ = 0.1546, *p* = 0.0025), and AST (R^2^ = 0.3529, *p* < 0.0001) (sFig. 1A-D). While there was a marginal correlation with CK (R^2^ = 0.0745, *p* = 0.0418), this association was not significant after removing outliers (R^2^ = 0.0339, *p* = 0.1872). Subgroup analysis by gender revealed a significant association between CK and ASM in female DM patients (R^2^ = 0.1342, *p* = 0.0185) but not in male DM patients (R^2^ = 0.0013, *p* = 0.8927) (sFig. 1E-F). However, no significant correlations were found between serum levels of ASM and CKBM, CRP, HRCT score, CD4, CD8, or KL6 (data not shown). Overall, these findings suggest a positive correlation between serum levels of ASM and disease activity in DM patients.

The EAM mouse model is frequently used to replicate the disease phenotype of polymyositis and DM in human. This model is commonly utilized to study the underlying pathological mechanisms and potential treatment targets of DM. In this study, we successfully established the EAM model and observed several key indicators of the successful model creation. When compared to the control mice, the EAM mice exhibited reduced muscle strength, increased serum CK levels, enlarged spleens, aggregated inflammatory cell infiltration, and higher histological scores on H&E staining (sFig. 2A-F). These findings suggest the successful establishment of the EAM model. Additionally, we assessed the expression levels of ASM in mouse muscle using qRT-PCR and IHC, as well as ASM activity. The results revealed that EAM model mice had higher mRNA and protein levels of ASM compared to control mice, along with enhanced ASM activity in the muscle (sFig. 3A-E). In summary, our findings demonstrate increased levels of ASM in the serum and muscle tissue of DM patients, accompanied by enhanced enzymatic activity. Furthermore, these elevated levels were positively correlated with disease activity.

### ASM involves in the pathology of EAM mice

**Fig. 2:**
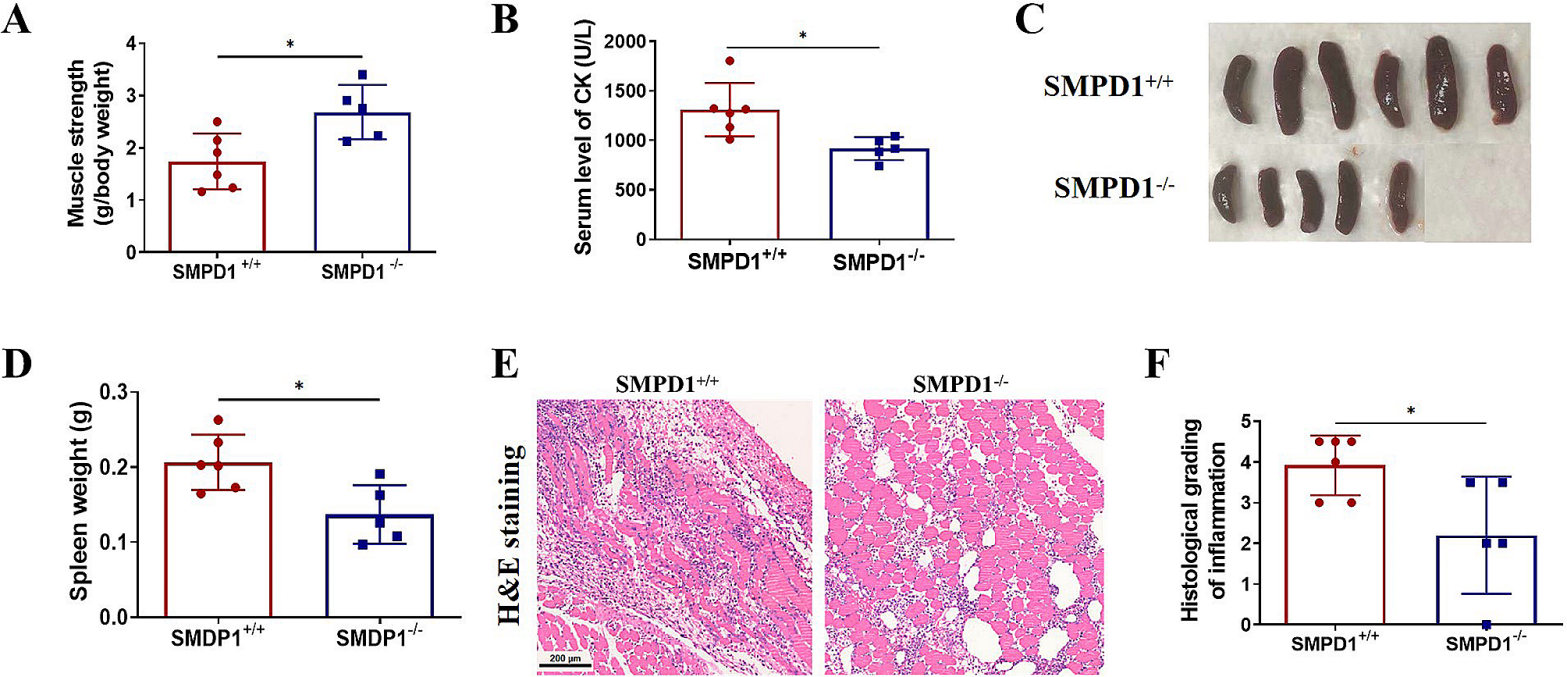
ASM plays a crucial role in the pathology of EAM mice. Establishment of EAM mouse model in ASM knockout (SMPD1^−/−^) mice. (**A**) muscle strength (**B**) serum CK level (**C**) spleen (**D**) spleen weight (**E**) H&E staining of muscle section (**F**) quantitative analysis of H&E staining. *P < 0.05, ** *P* < 0.01, *** *P* < 0.001, *n* = 6 in SMPD1^+/+^ mice group, *n* = 5 SMPD1^−/−^ mice group

Levels and activity of ASM were found to be elevated in both DM patients and EAM model mice. To investigate the significance of ASM in the pathology of the EAM mouse model, ASM gene knockout mice (SMPD1^−/−^) were utilized.The results demonstrated that SMPD1^−/−^ EAM mice exhibited higher higher muscle strength, reduced serum CK levels, decreased spleen size and weight, as well as reduced inflammatory cell infiltration and pathological score (Fig. 2A-F). These findings indicate that the disease severity in SMPD1^−/−^ EAM mice was alleviated, suggesting a potential role of ASM in the pathology of EAM.

### Amitriptyline is therapeutic against EAM mice model

**Fig. 3:**
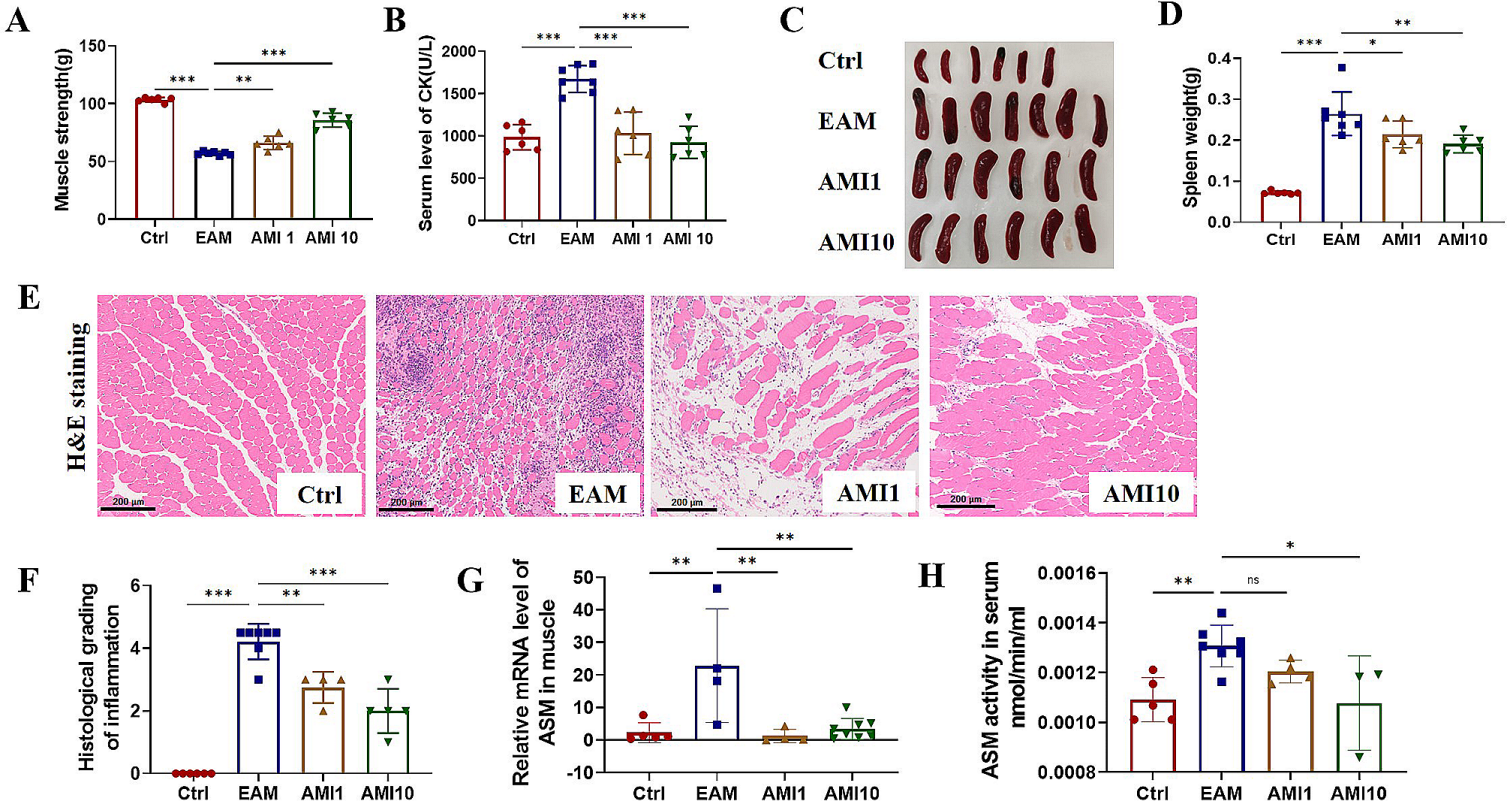
Amitriptyline is therapeutic against EAM mice model. To establish the EAM mouse model, wild type mice were subject to induction and subsequently treated with amitriptyline (AMI) at dosage levels of 1 mg/kg or 10 mg/kg. (**A**) muscle strength (**B**) serum CK level (**C**) spleen (**D**) spleen weight (**E**) H&E staining of muscle section (**F**) quantitative analysis of H&E staining. (**G**) mRNA expression level of ASM in muscle tissues (**H**) serum ASM activity. *P < 0.05, ** *P* < 0.01, *** *P* < 0.001, n ranges from 3 to 7 in each analysis group

Our previous results have highlighted the role of ASM in the pathology of the EAM mice model. To investigate the potential therapeutic effect of small molecule drugs targeting ASM on EAM mouse model, we selected AMI, a widely used ASM small molecule inhibitor. AMI was orally administrated at both higher (10 mg/kg) and lower (1 mg/kg) doses, starting two days before model induction. Results showed that compared to the control group, mice in the EAM model group exhibited decreased muscle strength, elevated serum CK levels, increased spleen weight, and increased muscle histopathology scores, indicating successful EAM model establishment (Fig. 3A-F). Treatment with both 1 mg/kg and 10 mg/kg doses of AMI significantly increased muscle strength, decreased serum CK levels, reduced spleen weight, and lowered muscle histopathology scores compared to the EAM model group (Fig. 3A-F). Furthermore, analysis of ASM mRNA expression via qRT-PCR and ASM activity in muscle tissue confirmed that AMI effectively suppressed both ASM expression and activity (Fig. 3G-H). These findings suggest that targeting ASM with the inhibitor AMI can alleviate disease severity in EAM model mice.

### The proportion of CD4 T cell subsets was imbalanced in EAM mice model

**Fig. 4:**
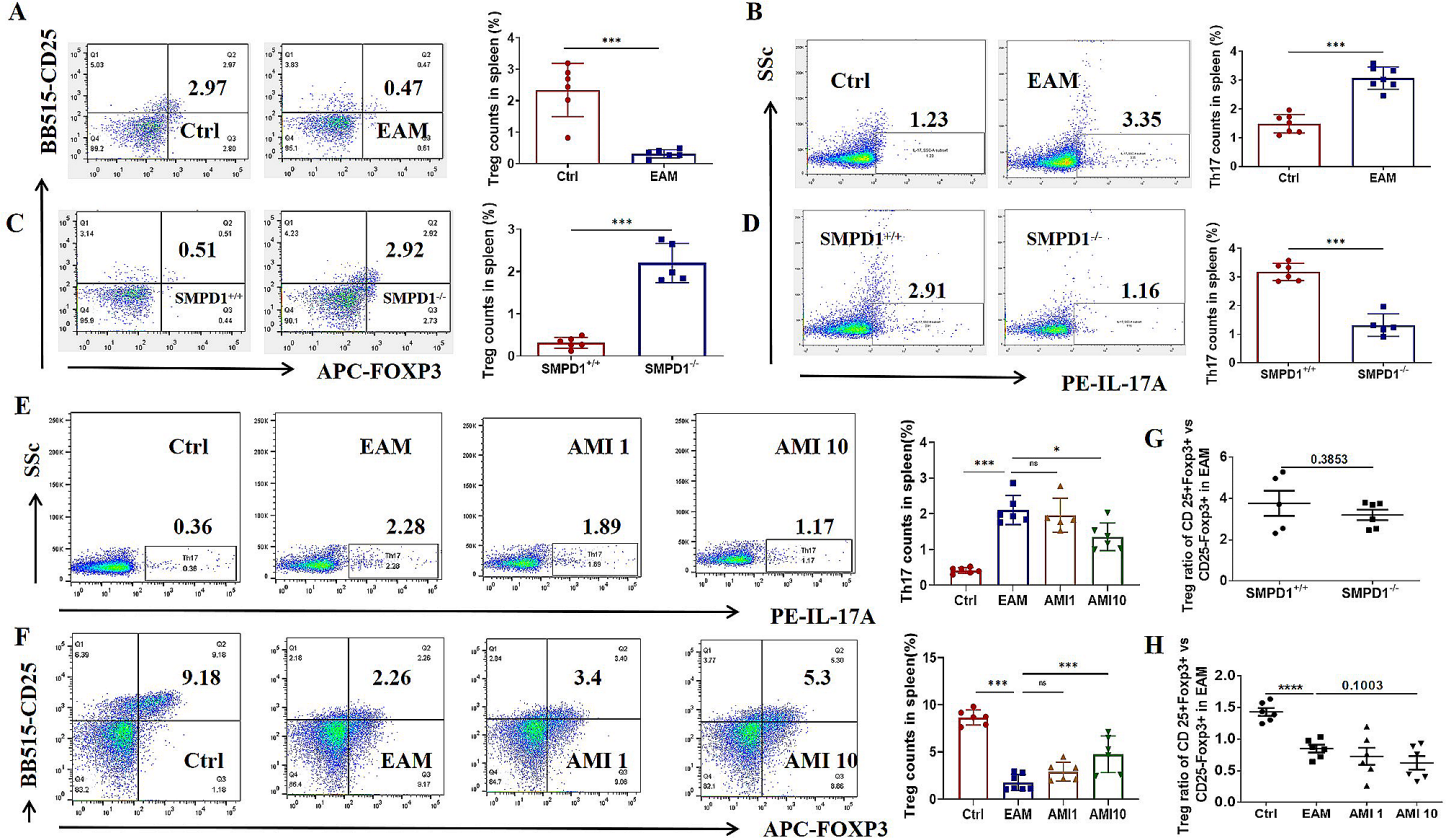
The proportion of CD4 T cell subsets was imbalanced in EAM mice model. Flow cytometry was applied to test the CD4 T cell subsets Treg and Th17 in spleen. (**A**) Treg in the wild type EAM model mice (**B**) Th17 in the wild type EAM model mice (**C**) Treg in the SMPD1^−/−^ EAM model mice (**D**) Th17 in the SMPD1^−/−^ EAM model mice (**E**) Th17 in the wild type EAM model mice treated with amitriptyline (**F**) Treg in the wild type EAM model mice treated with amitriptyline (**G**) Treg ratio of CD25 + Foxp3 + versus CD25-Foxp3 + in SMPD1^−/−^ EAM model mice (**H**) Treg ratio of CD25 + Foxp3 + versus CD25-Foxp3 + in EAM model mice treated with amitriptyline. *P < 0.05, ** *P* < 0.01, *** *P* < 0.001, n ranges from 5 to 7 in each analysis group

According to our study, ASM plays a critical role in the pathology of myositis. Inhibiting ASM activity with AMI has shown therapeutic effects in an EAM mouse model, prompting further investigation into the involvement mechanism of ASM in myositis pathology. Previous research has reported imbalances in CD4 T cell subsets in patients with DM, including a decrease in Treg cells and an increase in Th17 cells [28]. Restoring the balance of these CD4 T cell subsets may have therapeutic benefits. ASM has been identified as a key regulator in the differentiation of naive CD4 T cells [15]. Our findings in the EAM mouse model revealed similar results, showing an increase in Th17 cells and a decrease in Treg cells in the spleen compared to the control group (Fig. 4A-B). To investigate the role of ASM in regulating of naive CD4 T cell differentiation, we used SMPD1^−/−^ mice to establish the EAM model. The results demonstrated that knocking out ASM could rebalance the CD4 T cell subsets, increasing Treg cells and decreasing Th17 cells (Fig. 4C-D). To confirm whether the therapeutic effects of AMI are also achieved through regulating CD4 T cell subsets by affecting ASM, we performed flow cytometry analysis on spleen cells. The results indicated that AMI could reduce the increased Th17 cells and increase the reduced Treg cells (Fig. 4E-F). Furthermore, we analyzed the Treg ratio of CD25 + Foxp3 + versus CD25-Foxp3 + in EAM models, the data indicated a decreasing trend in the Treg ratio after knocking out SMPD1 or inhibiting ASM activity with amitriptyline (Fig. 4G-H).

Further, we examined the levels of cytokines IL-17 A, IL-10, and IL-6, which are known to impact the differentiation of Th17 cells [29]. Out results showed a significant increase in both mRNA expression and secretion levels of IL-17 A in the muscle tissue, spleen tissue, and serum of the EAM mouse model. Treatment with AMI was found to reduce the production of IL-17 A (sFig. 4A-D). Conversely, both mRNA expression and secretion levels of IL-10 were elevated in the muscle tissue of EAM mouse, with further enhancement upon AMI treatment (sFig. 4E-H). Similarly, both mRNA expression and secretion levels of IL-6 were elevated in the muscle tissue and serum of EAM mice, but decreased with AMI treatment(sFig. 4I-K). These findings indicate an imbalance in CD4 T cell subsets in the EAM mouse model, suggesting that targeting ASM could potentially rebalance these subsets.

### AMI regulates the CD4 T cells differentiation in EAM mouse model

**Fig. 5:**
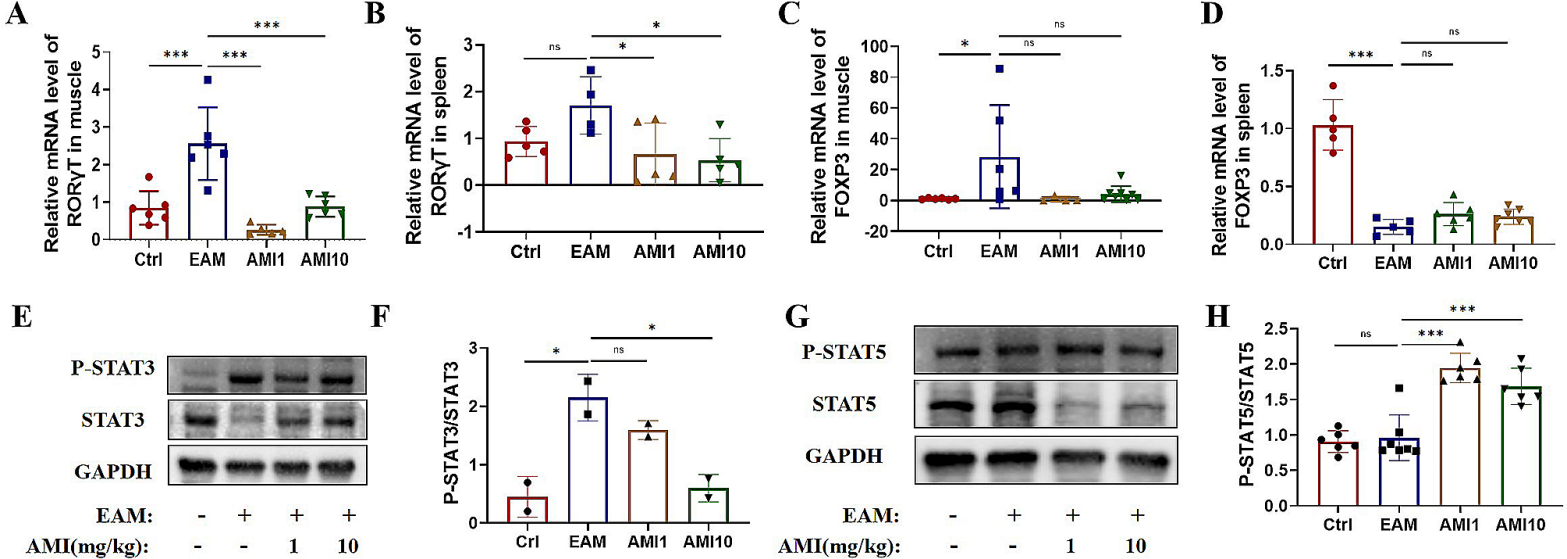
AMI regulates the CD4 + T cells differentiation in EAM mouse model. (**A**) mRNA expression level of RORγT in muscle tissues detected by qRT-PCR (**B**) mRNA expression level of RORγT in spleen detected by qRT-PCR (**C**) mRNA expression level of FOXP3 in muscle tissues detected by qRT-PCR (**D**) mRNA expression level of FOXP3 in spleen detected by qRT-PCR. (**E**) protein level of p-STAT3 and STAT3 in muscle tissue detected by WB (**F**) the quantitative anaysis of p-STAT3/STAT3 (**G**) protein level of p-STAT5 and STAT5 in muscle tissue detected by WB (**H**) the quantitative anaysis of p-STAT5/STAT5. *P < 0.05, **P < 0.01. ***P < 0.001, n ranges from 4 to 7 in each analysis group

The differentiation of naive CD4 + T cells into subsets is regulated by the relative transcription factors, RAR-related orphan receptor (RORγT) and FOXP3, which respectively regulate Th17 and Treg differentiation. Phospho-signal transducer and activator of transcription 3 (p-STAT3) is the key regulator for Th17, while phospho-signal transducer and activator of transcription 5 (p-STAT5) is the key regulator for Treg [29]. To evaluate these mechanisms, we analyzed the mRNA expression levels of transcription factors RORγT and FOXP3 in muscle and spleen tissues, as well as the protein levels of p-STAT3 and p-STAT5 in muscle tissues. The results showed an increase in both RORγT and FOXP3 mRNA expression in the muscle and spleen tissues of the EAM mouse group. However, AMI treatment only reduced the mRNA expression levels of RORγT (Fig. 5A-D). Additionally, the ratio of p-STAT3 to STAT3 was significantly higher in the EAM mouse group, but AMI reduced this ratio. Whereas, the ratio of p-STAT5 to STAT5 was not elevated in the EAM mouse group, but AMI increased this ratio (Fig. 5E-H). In summary, RORγT transcription factor and p-STAT3 protein level were increased in the EAM mouse group, and AMI effectively decreased their levels.

### AMI regulates the naive CD4 + T cell differentiation into Th17 in vitro

**Fig. 6:**
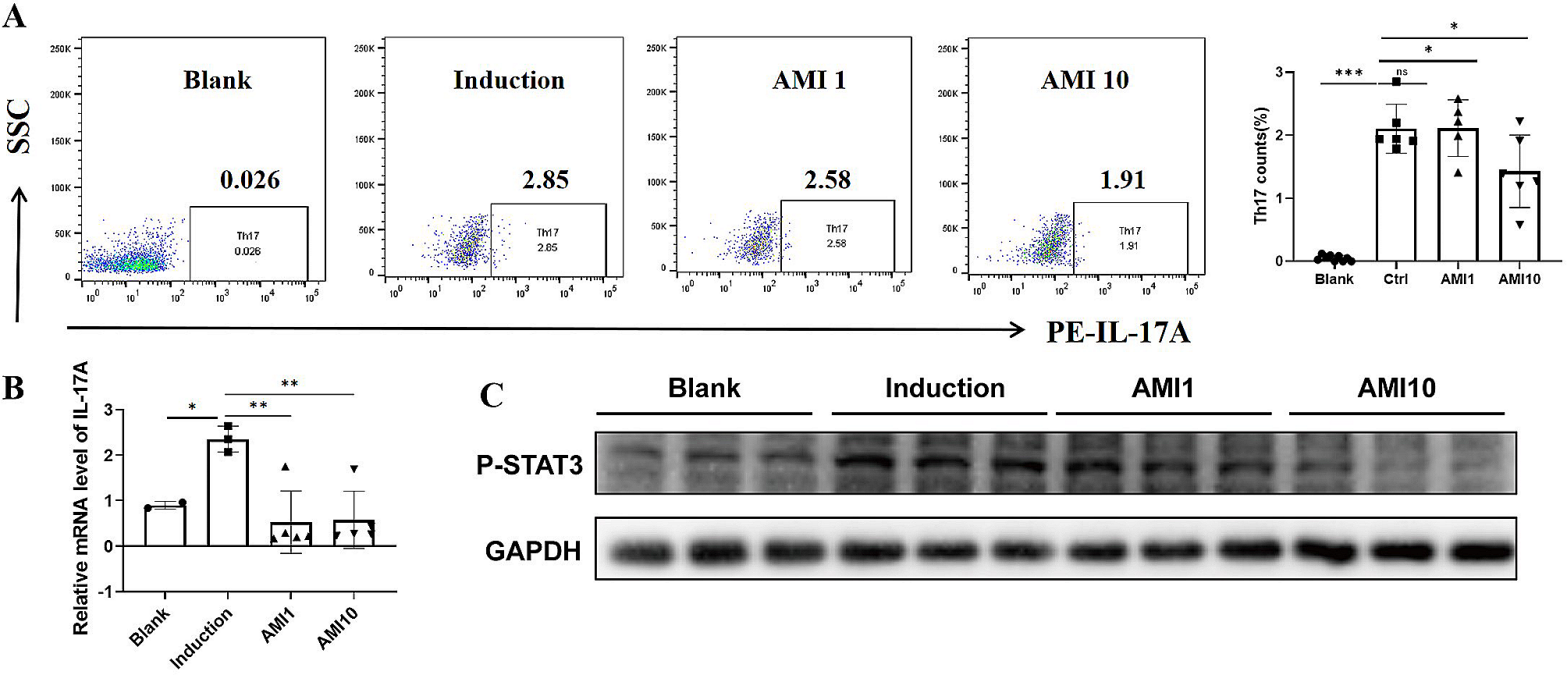
AMI regulates the naive CD4 + T cell differentiation into Th17 in vitro. Naive CD4 + T cells were isolated from spleen and then were induced to differentiate into Th17 under the Th17 differentiation condition. (**A**) Th17 subset detected by flow cytometry (**B**) mRNA expression level of IL-17 A detected by qRT-PCR (**C**) protein level of p-STAT3 detected by WB. *P < 0.05, **P < 0.01. ***P < 0.001, experiments were repeated twice

Our in vivo study demonstrated that ASM can affect the differentiation of CD4 + T cells. To further validate this finding, we isolated naive CD4 + T cells from the spleen and induced them to differentiate into Th17 subsets. Subsequently, we performed flow cytometry, mRNA analysis, and WB to assess the outcomes. The results showed that naive CD4 + T cells were effectively induced into Th17 subsets, showing a significant increase in IL-17 A mRNA expression level and an increase in p-STAT3 protein level in vitro. The treatment with AMI reduced the proportion of Th17 cells, decreased IL-17 A mRNA expression level, and lowered p-STAT3 protein levels (Fig. 6A-C). In summary, AMI can regulate the differentiation of naive CD4 + T cells into Th17 cells in vitro.

## Discussion

The pathology of DM is complex and remains incompletely understood, with current evidence suggesting the presence of immune cell abnormalities in tissues and circulation, involving various cell types such as T cells, macrophages, dendritic cells, B cells, mast cells and neutrophil [30, 31]. Immune imbalance is believed to be a key factor in the disease progression [32]. Both innate immunity and adaptive immunity are involved in the development of DM, with lymphocytes and their secreted cytokines playing a central role in the pathological mechanism. T lymphocytes, particularly the CD4 + T cells presented by MHC Class II peptide are the predominant cell types in the inflammatory infiltration of DM [33], contributing to its pathogenesis. This includes helper T cells Th1, Th2, and Th17, which secrete multiple cytokines to coordinate the immune response and provide co-stimulatory signals for antibody production. Additionally, Tregs inhibit the lytic activity of myoreactive CD8 + T cells and play an anti-inflammatory role [34].

In the context of DM, there is an imbalance in T cell subpopulations. Various studies have demonstrated that DM patients exhibit higher levels of Th17 cells and the cytokine IL-17 A in their muscle tissue [35, 36], as well as elevated serum levels of IL-17 A compared to healthy individuals. Moreover, the level of IL-17 A is positively correlated with disease activity [37]. Conversely, the number of Treg cells in the peripheral blood of DM patients is decreased, along with reduced levels of serum IL-10 [28, 38]. Th17 cells play a crucial role in maintaining the immune response and act as major regulators in the inflammatory microenvironment of muscle tissue [28]. On one hand, IL-17 produced by Th17 cells stimulates the production of IL-6 and chemokine 20, while also regulating the survival and differentiation of B lymphocytes [35]. On the other hand, IL-17 induces NF-κB activation, which inhibits myocyte migration and myogenic differentiation [39]. IL-6, a proinflammatory molecule in the inflamed microenvironment, is initially produced by innate immune responses such as macrophages. It then plays a role in the adaptive immune response by promoting CD4 + T differentiation into Th17 cells. IL-17, produced by Th17 cells, in turn, stimulates the production of IL-6, creating a mutual promotion effect [40, 41]. Tregs are a heterogeneous population, with those residing in muscle tissue capable of suppressing the inflammatory response in affected muscles, promoting the conversion of pro-inflammatory macrophages into an anti-inflammatory phenotype, and regulating the differentiation of muscle stem cells through the secretion of amphiregulin [42]. The delicate balance between pro-inflammatory T cell subsets and regulatory T cells is crucial for maintaining peripheral tolerance [28]. Therefore, correcting the immune imbalance of T cell subsets may represent a crucial therapeutic approach for managing DM. In our study, we observed an imbalance of T cell subsets in EAM model mice, specifically an increase in Th17 cells and their secretion of IL-17 A and the inflammatory cytokine IL-6, alongside a decrease in Treg cells and their secretion of IL-10.

According to previous reports, ASM plays a crucial role in regulating the differentiation of naive CD4 + T cells into T cell subsets. ASM accomplishes this by mediating CD3 and CD28 signal transduction through ceramide production, thereby controlling the activation and proliferation of naive CD4 + T cells. This process facilitates the differentiation of Th1 and Th17 cells, while simultaneously impeding the quantity and function of Treg cells [15, 43]. In the differentiation process of Th17 cells, the key molecule is STAT3, whereas STAT5 is important in Treg differentiation. Upon exposure to TGF-β and IL-6, STAT3 is activated in conjunction with TCR-co-stimulative signals. The phosphorylation of STAT3 triggers the expression of the transcription factor RORγt, which fosters the differentiation of CD4 + T cells into Th17 cell subpopulations. Simultaneously, the differentiation of Treg cells is hindered by the down-regulation of TGF-β-induced FOXP3 [44]. Conversely, IL-2 signaling triggers the phosphorylation of STAT5, leading to its binding to the FOXP3 promoter and subsequent induction of FOXP3 expression [45]. Furthermore, TGF-β can activate of Sma- and Mad-related protein (SMAD) 2 and SMAD3 [46], which in turn stimulate the transcription factor FOXP3 and facilitate the differentiation of Treg cells from naive CD4 + T cells.

In our study, we examined the activity and levels of ASM in both DM patients and EAM model mice. We observed elevated ASM activity and serum levels in DM patients. Subgroup analysis based on gender did not show a statistical difference in serum ASM levels, but a correlation between serum ASM levels and CK levels was found in female DM patients rather than male patients, possibly due to gender difference [47]. However, further research is needed due to the limted sample size of enrolled population. In EAM model mice, there was an increase in ASM activity and protein levels inmuscle tissues. The CD4 + T cell subsets Th17 showed an increase, while Treg levels were reduced. Interestingly, in SMPD1^−/−^ knockout EAM model mice, we observed a reversal of the imbalance between Th17 and Tregs, leading to a reduction in disease severity. Moreover, treatment with the ASM inhibitor amitriptyline in the EAM model mice resulted in a therapeutic effect by balancing Th17 and Treg subsets. These findings suggest that ASM plays a crucial role in regulating the differentiation of naive CD4 + T cells into T cell subsets, and targeting ASM could potentially reduce EAM severity. Further investigations revealed that ASM can regulate the phosphorylation levels of STAT3 and STAT5 in vitro, indicating its involvement in regulating the differentiation of CD4 + T cells into Th17 and Treg subsets by controlling key protein molecules’ phosphorylation Levels.

Compared to the EAM model group, treatment with AMI resulted in a reduction in IL-6 expression. The varying levels of IL-6 played a crucial role in creating inflammatory environments that influenced the differentiation of naive CD4 + T cells into Th17 cells or Treg cells. The administration of AMI in EAM mouse models led to decreased expression of RORγT in the spleen and muscle, as well as reduced levels of phosphorylated STAT3 protein in muscle tissue. This suggested that targeting ASM could inhibit the differentiation of CD4 + T cells into Th17 cells. However, the levels of IL-10 and the transcription factor FOXP3 did not consistently change across different tissues. In the spleen of the EAM model group, the expressions of IL-10 and FOXP3 were lower compared to the control group, while in muscle tissue, IL-10 and FOXP3 levels were higher than in serum. Although AMI treatment increased IL-10 levels in EAM mice, it did not increase the level of FOXP3. Burzyn et al. proposed that Treg cells in injured muscle tissue represent a distinct subtype of CD4 + CD25 + FOXP3 + Treg cell population, exhibiting a unique transcriptome and TCR pool compared to those in lymphoid organs [48]. Previous studies have shown that Treg cells are more abundant in the muscles of adolescents with DM compared to normal muscles [49, 50]. Similarly, adult DM muscle biopsies have also shown a high presence of Treg cells [51], indicating that the increase in Treg cells is a response to local muscle inflammation. While the specific mechanism behind the enrichment of Treg cells in inflammatory muscles remains incompletely understood, previous studies [52, 53] have demonstrated that the increased migration of Treg cells to inflamed muscles can mitigate muscle injury and promote the repair of chronically inflamed muscles. This beneficial effect is linked to effector molecules produced by Treg cells, such as IL-10, which has been associated with a protective role in muscle dystrophy [54, 55] and the promotion of muscle repair following loss injury [56]. Therefore, the enrichment of Treg cells and their effector molecules, including IL-10, in local muscles plays a crucial role in facilitating the repair of injured muscles. In line with these findings, our study observed a significant increase in levels of the anti-inflammatory factor IL-10 after treating EAM mouse models with the ASM inhibitor AMI, leading to a marked reduction in disease severity and muscle damage.

The Treg cells present at the inflamed sites exhibit instability and plasticity [57]. Alongside the CD4 + CD25 + Foxp3 + T cells, the subgroup of CD4 + CD25-Foxp3 + T cells has also been implicated in various immune dysregulatory diseases, such as systemic lupus erythematous. In this context, the proportions of CD4 + CD25-Foxp3 + T cells have shown a positive association with disease activity markers like dsDNA, complements, proteinuria and renal involvement. Conversely, these proportions tend to decrease as the disease transitions into an inactive state [58]. Our analysis of the Treg ratio between CD25 + Foxp3 + and CD25-Foxp3 + in EAM model revealed a decreasing trend in the Treg ratio following the knockout of SMPD1 or inhibition of ASM activity. The potential role of CD25-Foxp3 + T cells in EAM pathology and the involvement of ASM in regulating CD25 expression and Treg subsets warrant further investigation.

## Conclusion

ASM plays a crucial role in the pathology of DM by regulating the transformation of naive CD4 + T cells into Th17 and Treg subsets. This not only offers a promising therapeutic target for DM but also enhances comprehension of how amitriptyline functions as a treatment. Furthermore, it introduces novel opportunities for utilizing amitriptyline in conditions linked to increased ASM activity.

### Electronic supplementary material

Below is the link to the electronic supplementary material.

Supplementary Material 1

## Data Availability

No datasets were generated or analysed during the current study.
