# Supplementary material for: ASM is a therapeutic target in dermatomyositis by regulating the differentiation of naive CD4 + T cells into Th17 and Treg subsets
Source: Skelet Muscle. 2024 Jul 18;14:16. doi: 10.1186/s13395-024-00347-1 (PMC11256435; doi:10.1186/s13395-024-00347-1)
Supplement: Supplementary file 1 — Supplementary Material 1 [file 13395_2024_347_MOESM1_ESM.docx]

**ASM is a therapeutic target in dermatomyositis by regulating the differentiation of naive CD4+T cells into Th17 and Treg subsets**

Yuehong Chen^1^, Huan Liu^1^, Zhongling Luo^1^, Jiaqian Zhang^1^, Min Dong^1^, Geng Yin^2^, Qibing Xie^1^

Yuehong Chen and Huan Liu contributed equally to this study.

^1^Department of Rheumatology and Immunology, West China Hospital, Sichuan University, Chengdu 610041, China

^2^Department of General Practice, General Practice Medical Center,West China Hospital, Sichuan University, Chengdu 610041, China

*** Correspondence:**

Geng Yin, email: yingeng1975@163.com, Department of General Practice,General Practice Medical Center,West China Hospital,Sichuan University, 37 Guoxue lane, Chengdu 610041, China, Tel/Fax: +86-28-8542 2393

Qibing Xie, email: xieqibing1971@163.com, Department of Rheumatology and Immunology, West China Hospital, Sichuan University, 37 Guoxue lane, Chengdu 610041, China, Tel/Fax: +86-28-8542 2393

Supplemental figures: sFig.1-sFig.4


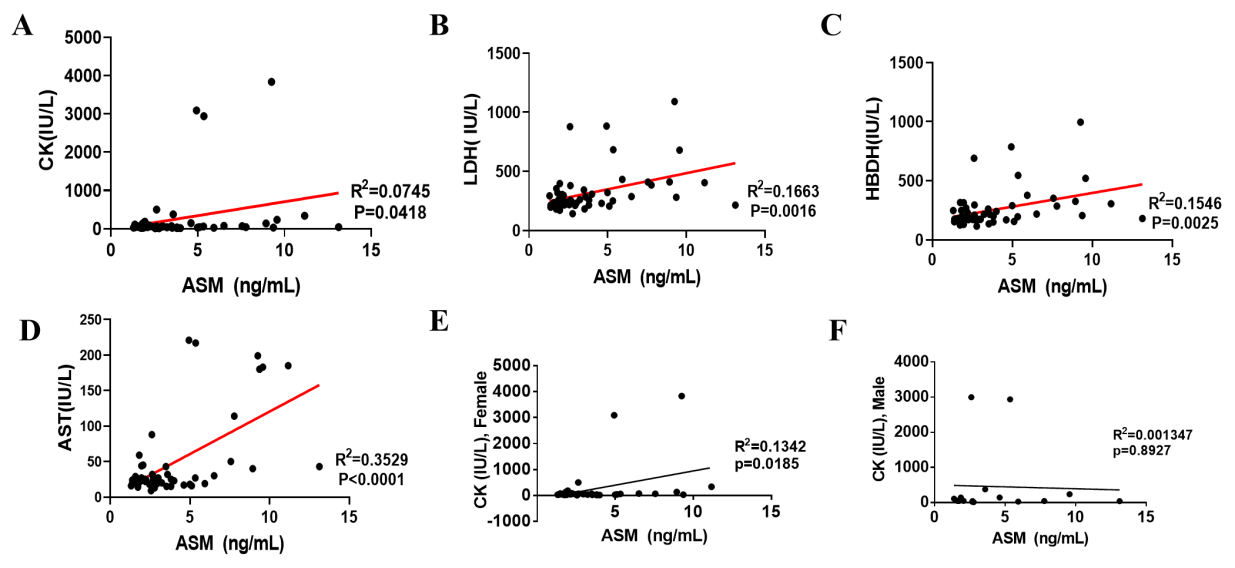


sFig.1 Correlation analyses between serum ASM levels and muscle enzyme levels in DM patients. (A) Correlation between ASM and CK level (B) Correlation between ASM and LDH level (C) Correlation between ASM and HBDH level (D) Correlation between ASM and AST level (E) Correlation between ASM and CK level in female DM patients (F) Correlation between ASM and CK level in male DM patients. *P<0.05, ** P<0.01, *** P<0.001.


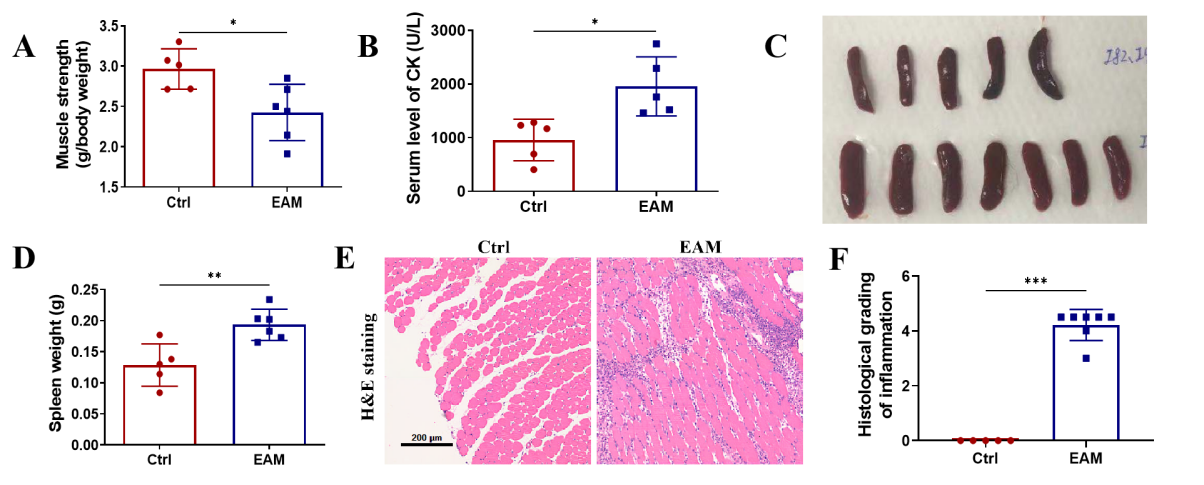


sFig.2 Establishment of EAM mouse model in wild type mice. (A) muscle strength (B) serum CK level (C) spleen (D) spleen weight (E) H&E staining of muscle section (F) quantitative analysis of H&E staining . *P<0.05, ** P<0.01, *** P<0.001.


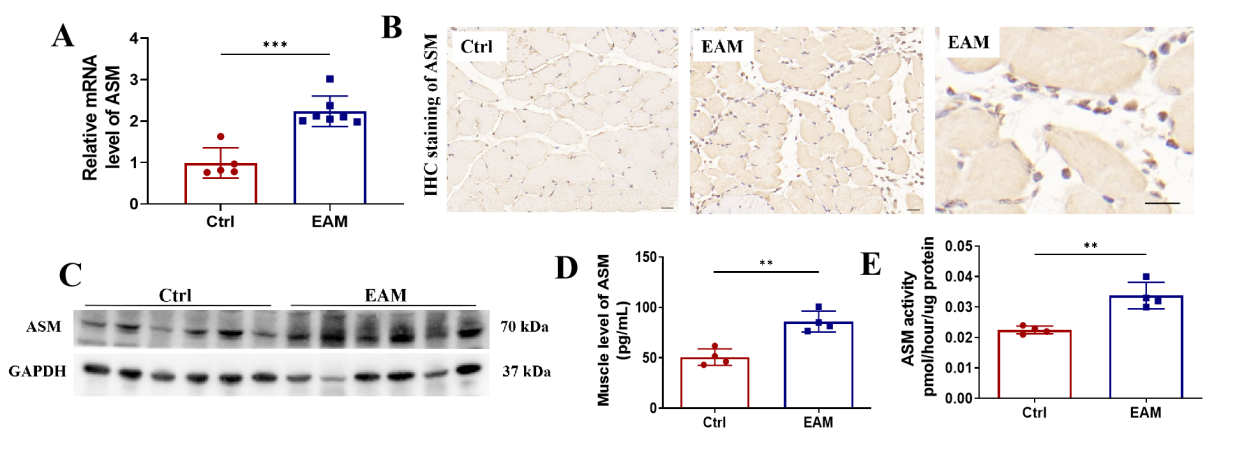


sFig.3 Level and activity of ASM in muscle specimens of EAM mice. (A) the mRNA expression levels of ASM detected by qRT-PCR (B) the protein level of ASM detected by IHC, scale bar 20 μm (C) the protein level of ASM tested by WB (D) the expression level of ASM detected by ELISA (E) the activity of ASM. *P<0.05, ** P<0.01, *** P<0.001.


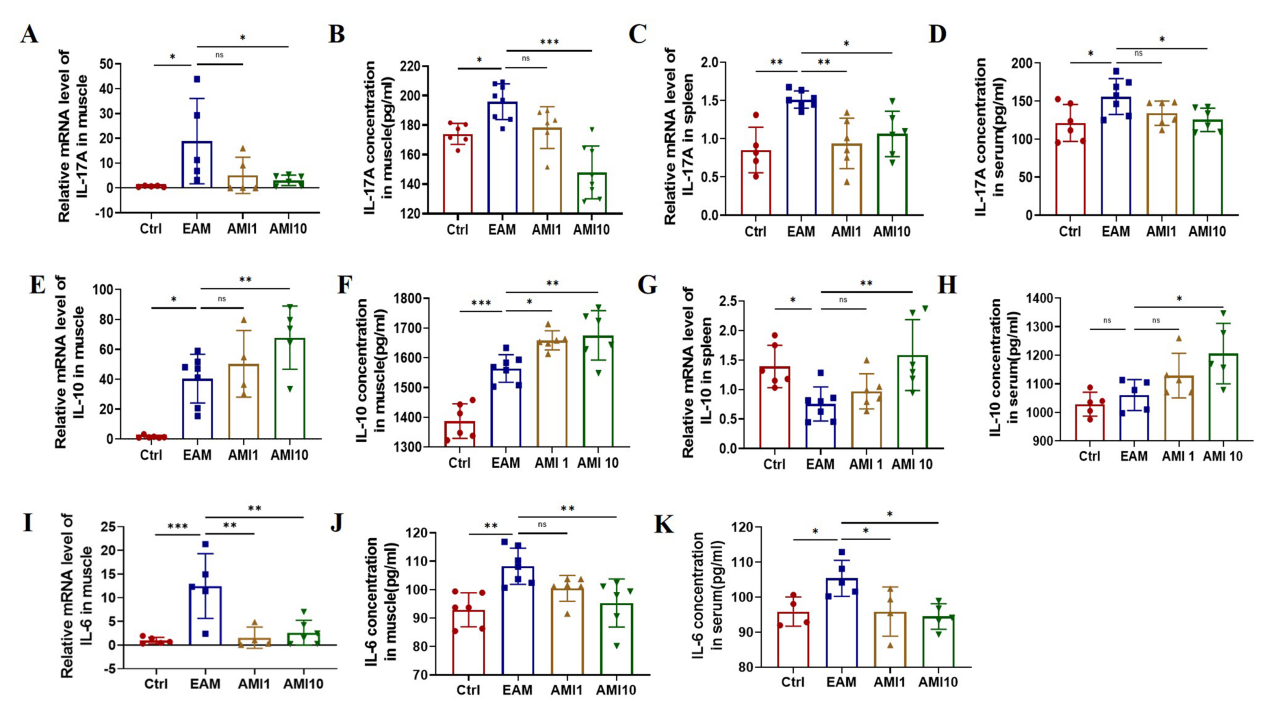


sFig.4 AMI reduces the production of cytokines IL-6 and IL-17A while increases IL-10 in EAM mice model. (A) mRNA expression level of IL-17A in muscle tissues detected by qRT-PCR (B) protein level of IL-17A in muscle tissues detected by ELISA (C) mRNA expression level of IL-17A in spleen detected by qRT-PCR (D) serum level of IL-17A detected by ELISA (E) mRNA expression level of IL-10 in muscle tissues detected by qRT-PCR (F) protein level of IL-10 in muscle tissues detected by ELISA (G) mRNA expression level of IL-10 in spleen detected by qRT-PCR (H) serum level of IL-10 detected by ELISA (I) mRNA expression level of IL-6 in muscle tissues detected by qRT-PCR (J) protein level of IL-6 in muscle tissues detected by ELISA (K) serum level of IL-6 detected by ELISA. *P<0.05, **P<0.01. ***P<0.001.
